# Harnessing the Radical Reactivity of Silver(II) Fluoride for Organofluorine Synthesis

**DOI:** 10.21203/rs.3.rs-7216247/v1

**Published:** 2025-08-04

**Authors:** Sudip Maji, Suchand Basuli, Subhrashis Banerjee, Deepak R. Pradhan, Jennifer S. Hirschi, Mathew J. Vetticatt

**Affiliations:** Department of Chemistry, Binghamton University, Vestal, NY 13850, USA

## Abstract

Most practical fluorination reagents deliver a fluorine atom either as a nucleophile (F^−^) or as an electrophile (F^+^). In contrast, bench-stable radical fluorine (F^•^) reagents are relatively less common and the vast majority of ‘radical fluorinations’ involve reactions of carbon-centered radicals with electrophilic fluorination reagents. Here, we disclose that silver (II) fluoride (AgF_2_) in acetonitrile is a mild source of F^•^ that can be leveraged for the synthesis of a variety of high-value organofluorine compounds from abundantly available reactants such as alkanes, alkenes, and carboxylic acids, as well as from pharmaceutically relevant heterocycles such as indoles and benzofurans. This platform technology obviates the need for expensive catalysts and fluorinating reagents that are typically necessary to accomplish these transformations and relies on the use of AgF_2_ in acetonitrile as the sole reagent under mild conditions.

## Introduction:

**Fig 1.**
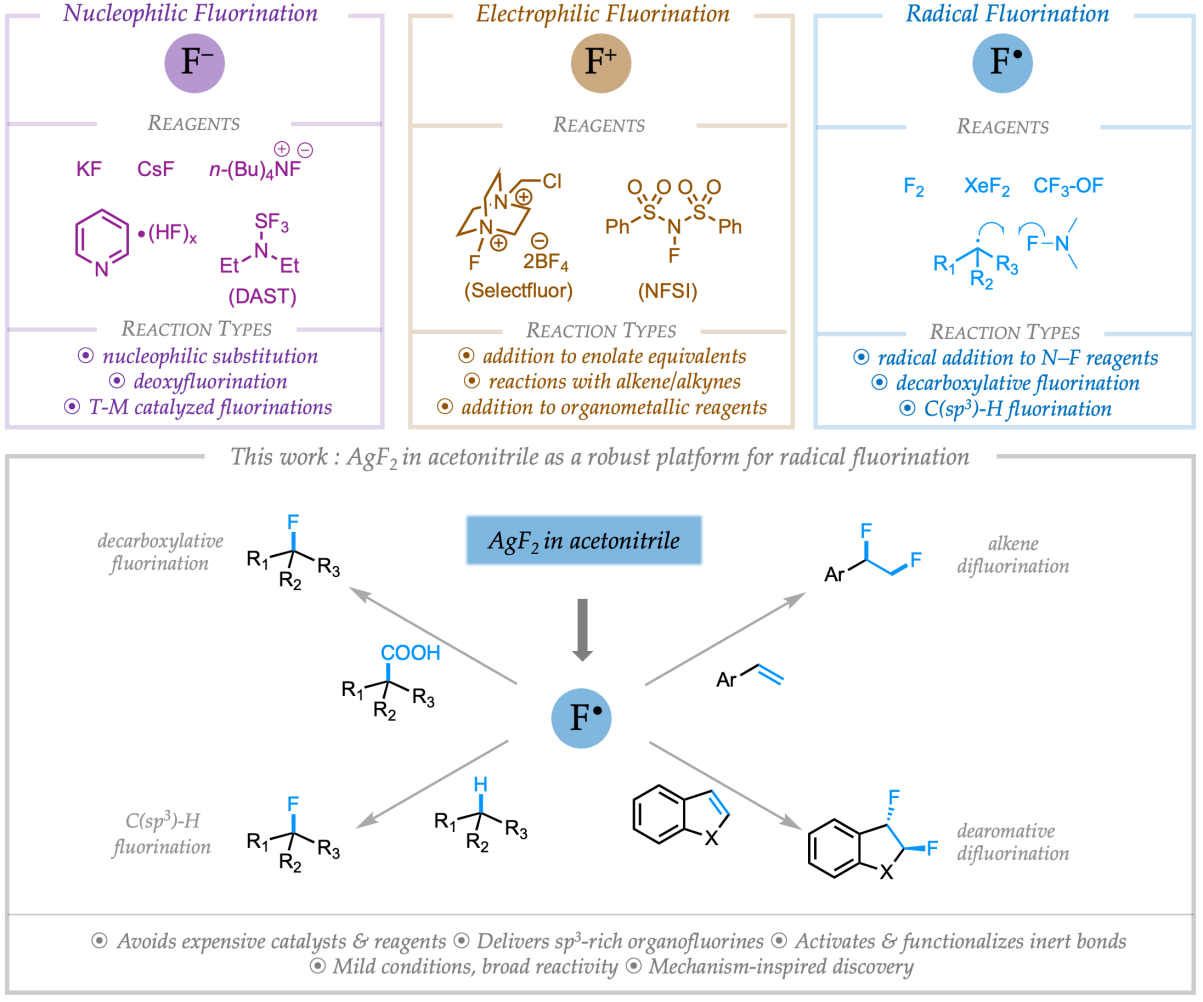
Complementary strategies and reagents used for the formation of C–F bonds. The current work establishes silver (II) fluoride as a general reagent for radical fluorinations.

The high electronegativity and small size of a fluorine atom imparts unique properties to molecules. It is well established that introduction of fluorine into drug molecules can enhance efficacy and metabolic stability.(1, 2) The importance of fluorine-containing targets in the pharmaceutical industry cannot be overstated – ~30% of drug molecules that received FDA approval in the past ten years contain at least one fluorine atom.(3, 4) Therefore, development of new, operationally trivial methods for the incorporation of fluorine in organic molecules will have a significant impact on drug discovery efforts in both academia and the pharmaceutical industry. The current state-of-the-art in C–F bond formation can broadly be classified into three categories depending on the electronic nature of the fluorine atom involved in the key step (Fig. 1). Nucleophilic fluorination reactions, where a fluoride ion (F^−^) acts as a nucleophile and forms a bond with an electrophilic carbon atom, are well developed with numerous inexpensive fluorinating agents available such as alkali metal fluorides and HF-based reagents.(5, 6) The second category involves the use of electrophilic fluorinating reagents (sources of F^+^) for reactions with nucleophilic carbon centers such as enolate equivalents or electron-rich alkenes. The most widely-used electrophilic fluorinating reagents are molecules containing an N–F bond such as Selectfluor^™^ or *N*-fluorosuccinamide (NFSI).(7, 8) Radical fluorination is relatively under-developed due to the hazardous nature of F^•^ reagents (eg. F_2_, XeF_2_, and hypofluorite). Consequently, most practical radical-based C–F bond-forming transformations proceed via reaction between a carbon-centered radical and electrophilic N–F fluorinating reagents, which have been shown to also behave as F^•^ transfer reagents.(9, 10) The development of a straightforward, broadly applicable, and robust radical fluorination methodology, involving reaction between a fluorine radical and closed-shell organic molecules, is arguably an unmet goal in organofluorine synthesis.

**Fig. 2.**
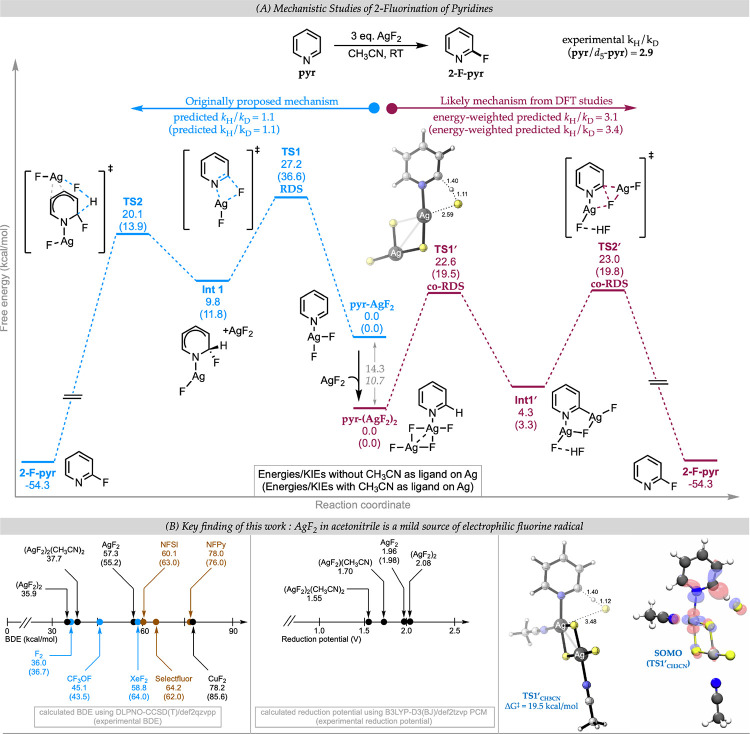
(A) DFT Investigation of the mechanism of 2-fluorination of pyridine reported by Hartwig using B3LYP-D3BJ/def2TZVP PCM(acetonitrile). Numbers in parenthesis are from calculations performed with an acetonitrile ligand on the silver atoms. The results suggest the involvement of an electrophilic fluorine radical in the key step (TS1´). (B) *Left panel –* calculated and experimental (in parenthesis) BDE for fluorinating reagents that participate in radical reactions. *Middle panel* – calculated and experimental reduction potential (in parenthesis) for AgF2 illustrating the role of acetonitrile in taming the oxidizing power of AgF2. *Right panel –* FMO analysis of the key H-atom abstraction transition state supports the finding that acetonitrile ligated AgF2 is a mild source of fluorine radicals.

Our research groups utilize a mechanism-guided approach for the discovery of novel reactivity in organic synthesis, a critical part of which focuses on investigation of the mechanisms of existing reactions to provide valuable clues that might aid in the design of more efficient reactions. As part of this research focus, we initiated a mechanistic study of a reaction reported by Hartwig and Fier involving the fluorination of pyridines using super-stoichiometric silver (II) fluoride (AgF_2_) in acetonitrile.(11, 12) These studies reveal a novel mechanism for this transformation, where acetonitrile ligated AgF_2_ acts as a mild ***source of electrophilic fluorine radical*** in the key step of the reaction (Fig. 2A). Based on this mechanistic finding and recognizing the potency of fluorine radicals to rapidly form C–F bonds and abstract hydrogen atoms from unreactive C–H bonds (due to the thermodynamic stability of the resulting C–F and H–F bonds, respectively), we sought to leverage these mild conditions to develop a platform technology for the synthesis of organofluorines by radical fluorination of abundant starting materials. We report herein, the successful realization of this strategy for the synthesis of sp^3^-rich organofluorine compounds from feedstock chemicals such as alkanes, alkenes, and carboxylic acids as well as from reactions with heterocyclic scaffolds such as indoles and benzofurans that are of broad interest to the pharmaceutical industry (Fig. 1).

## Mechanistic studies of 2-fluorination of pyridine:

In their original report of the AgF_2_ mediated fluorination of pyridine, Hartwig proposed a mechanism initiated by coordination of AgF_2_ to pyridine (Fig. 2, **pyr-AgF**_**2**_) followed by addition of the [Ag]-F bond across the p-system of the pyridine (**TS1**) to form an amido-silver(II)-fluoride complex (**Int1**). Rearomatization of this intermediate occurs via H-atom transfer (**TS2**) to another molecule of AgF_2_ to deliver 2-fluoropyridine and two equivalents of silver(I) fluoride (AgF). They measured a competitive *k*_H_/*k*_D_ of 2.9 (for pyridine v/s *d5*-pyridine, Fig. 2A) and reasonably interpreted this as evidence for the second step (TS2) being rate-determining. Our DFT investigation of the Hartwig mechanism (Fig. 2A, blue reaction coordinate) suggests that contrary to their proposal, **TS1** is the rate-determining step in this mechanism with a free energy barrier of 27.2 kcal/mol relative to the **pyr-AgF**_**2**_. The ensuing HAT step (**TS2**) is only 20.1 kcal/mol above the **pyr-AgF**_**2**_. If **TS1** is the rate-determining step of this reaction then the predicted *k*_H_/*k*_D_ for **TS1** of 1.1 is inconsistent with the experimental *k*_H_/*k*_D_ of 2.9 (Fig. 2B), indicating that this is likely not the mechanism of pyridine fluorination.

Further evaluation of alternative pathways reveals a fundamentally different pathway as the most likely mechanism for this reaction. Computations suggest that the complex of pyridine with two molecules of AgF_2_ (**pyr-(AgF**_**2**_**)**_**2**_) is likely the resting state complex. Subsequently, the more likely mechanism involves H-atom abstraction of the sp^2^ C–H by one of the fluorine atoms (**TS1´**). The resulting 2-pyridyl radical intermediate is a transient species that rapidly adds to one of the silver atoms. Reductive elimination from this complex (**TS2´**) delivers 2-fluoropyridine and two equivalents of silver(I) fluoride (AgF). The two transition structures **TS1´** (ΔG^‡^ = 22.6 kcal/mol) and **TS2´** (ΔG^‡^ = 23.0 kcal/mol) are relatively close in energy (Fig. 2B, maroon numbers). Intriguingly, the coordination of acetonitrile solvent as a ligand to the silver results in the lowering of the absolute barriers for both steps in this mechanism by 3.1 and 3.2 kcal/mol, respectively (Fig. 2B, italicized maroon numbers). These computed barriers are more consistent with a reaction that proceeds at room temperature and with the experimental observation that acetonitrile is required for reactivity. The predicted *k*_H_/*k*_D_ of 3.1 (3.4 for the model with acetonitrile as ligand) for this mechanism is in good agreement with the experimentally measured *k*_H_/*k*_D_ of 2.9 (Fig. 2A).

The computed BDEs for AgF_2_ and (AgF_2_)_2,_ with and without solvent coordination is shown in comparison to the computed BDEs of common radical fluorine reagents (Fig. 2B). The acetonitrile coordinated AgF_2_ dimer (AgF_2_)_2_(CH_3_CN)_2_ has a computed BDE of only 37.7 kcal/mol, which is similar to the computed BDE of F_2_ (36.0 kcal/mol), suggesting that a radical fluorine-based mechanism is viable. Intriguingly, coordination of acetonitrile to AgF_2_ or (AgF_2_)_2_ results in a significant decrease in the respective reduction potentials (Fig. 2B), suggesting that acetonitrile ligation makes silver (II) fluoride a milder oxidant. A comparison of the transition state structure of the H-atom abstraction step by AgF_2_ modeled with acetonitrile as a ligand (Fig. 2B, **TS1´**_**CH3CN**_) and without acetonitrile as ligand (Fig. 2A, **TS1´**) reveals similar distances for the breaking C–H bond and the forming H–F bond. However, the silver-fluorine distance is significantly more elongated in **TS1´**_**CH3CN**_ suggesting that acetonitrile facilitates Ag–F bond dissociation by stabilizing the silver center. Analysis of the frontier molecular orbitals of **TS1´**_**CH3CN**_ shows that the SOMO-HOMO gap (1.8 kcal/mol) in this transition state is significantly smaller than the SOMO-LUMO gap (48.2 kcal/mol) – further confirming the involvement of an electrophilic fluorine radical in the key step of this reaction. With the discovery that a fluorine radical is involved, we hypothesized that we could take advantage of these conditions to enable the types of reactions that are typically classified as radical fluorinations (Fig. 1). In the following sections, we describe the realization of this hypothesis in the form of a variety of novel radical fluorination reactions that will have a significant impact on the field of organofluorine synthesis.

## Fluorination of alkenes and alkynes:

The addition of fluorine across alkenes under mild conditions is a desirable transformation as it provides access to two fluorinated sp^3^ carbon centers from planar alkenes. State-of-the-art in alkene difluorination relies on the use of iodoarenes as catalysts, an excess of a fluoride source (typically amine:HF), and an external oxidant (Selectfluor, *m*CPBA).(13, 14, 15, 16) Under these conditions, the in-situ generated iodoarene difluoride plays the dual role of activating the alkene and generating a fluoride anion (F^−^⋯H-F). The activated alkene then undergoes consecutive nucleophilic substitution by the fluoride anion to deliver the difluorinated alkene and the iodoarene, which is reoxidized to iodoarene difluoride by an external oxidant (9–11) or electrochemically (12).

We envisioned that the high electrophilicity of F^•^, (17) generated by AgF_2_ in acetonitrile would enable direct addition to the olefin (**1**) to form a C–F bond and a transient carbon-centered radical. This intermediate would then either abstract a fluorine atom from another molecule of AgF_2_ to directly yield the difluorinated product (**2**) or form a C–Ag bond with AgF_2_ resulting in an Ag(III) intermediate, which could deliver **2** via reductive elimination. Alternatively, a mechanism might be envisioned where two molecules of AgF_2_ simultaneously deliver two fluorines to the alkene in a concerted fashion. The proposed transformation would generate vicinal difluorination products with AgF as the only side-product. The use of AgF_2_ would obviate the need for an iodoarene catalyst or an external oxidant and would be the mildest and most straightforward approach for the vicinal difluorination of alkenes.

**Fig. 3.**
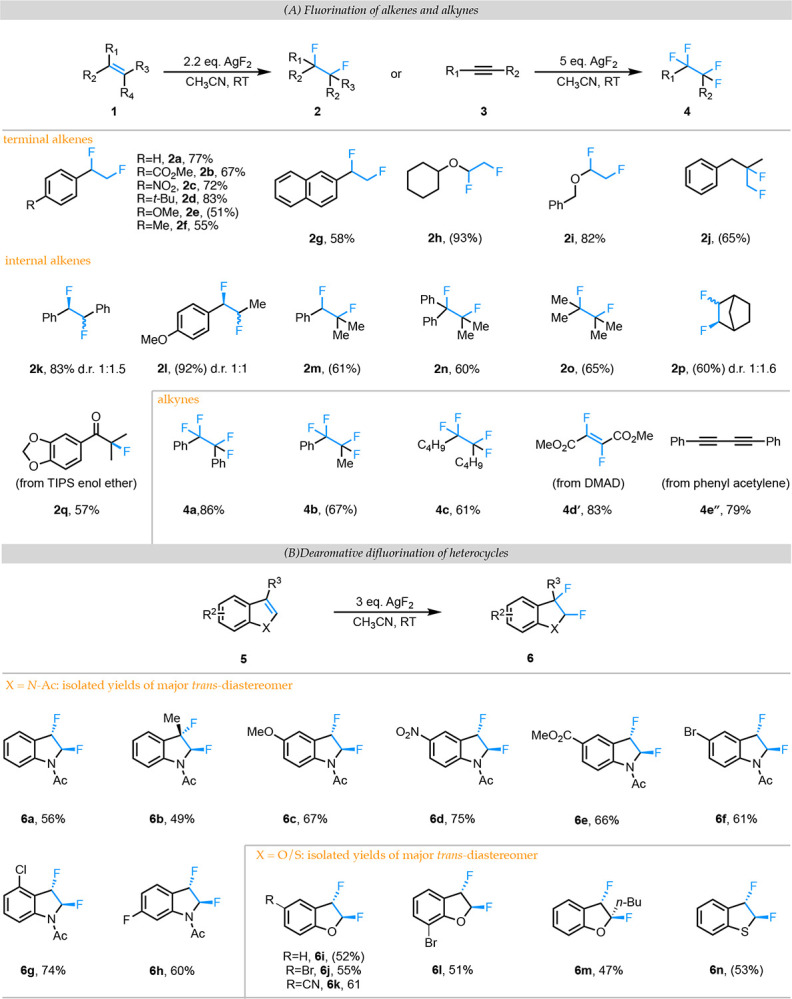
(A) Substrate scope for the fluorination of alkenes and alkynes using AgF2 as the sole fluorinating agent. (B) Substrate scope for the dearomative difluorination of heterocycles using AgF2 as the sole fluorinating agent. Crude d.r. in the reaction mixture ranged from 2.3:1 to 7.3:1 for indoles and 1.2:1 to 1.8:1 for the other heteroycles. The minor diasteromer was not isolated. (A&B) Yields in parenthesis are NMR yields based on inert internal standard added to the reaction mixture. All others are isolated yields.

We tested our hypothesis by subjecting a range of electronically and sterically diverse alkenes to 2.2 equivalents of AgF_2_ in acetonitrile (Fig. 3A). Under these conditions, electron-rich and electron-deficient *p*-substituted styrenes (**1a**–**g**) as well as di-,tri-, and tetra-substituted styrene derivatives (**1k**–**n**) gave the desired difluorinated alkenes (**2a**–**g** and **2k**–**n**) in good to excellent yields. Unactivated olefins were also viable substrates under these conditions, giving good to moderate yields of the difluorinated products (**2j**, **2o**, and **2p**). Electron-rich vinyl ethers such as benzyl vinyl ether and cyclohexyl vinyl ether gave excellent yields (>80%) of the corresponding products (**2h**, **2i**) while electron-poor alkenes such as β-nitrostyrene, dimethylfumarate, and ethyl acrylate did not yield any observable products (see Supporting Information). This result supports our mechanistic hypothesis that the reaction proceeds via addition of an electrophilic fluorine radical and possibly discounts the involvement of a nucleophilic fluoride ion as the fluorine source. Interestingly, the reaction of the TIPS protected silyl enol ether of an aromatic ketone proceeds exclusively to a-fluoro ketone **2q** rather than the expected difluorinated alkene. We also evaluated alkynes (**3**) as potential substrates for this reaction using 5.0 equivalents of AgF_2_ to achieve tetrafluorination. Under these conditions, internal alkynes delivered ~60–85% yield of the tetrafluorinated products (**4a**-**4c**). Internal alkynes flanked by electron-withdrawing groups (such as **3d**) stalled at difluorination, and the resulting difluorinated alkene product **4d´** was isolated in excellent yield exclusively as the *trans*-isomer. Presumably, **4d´** is too electron-poor to participate in further fluorination by an electrophilic fluorine radical. Intriguingly, phenylacetylene (a terminal alkyne) did not yield the expected tetrafluorinated product. Instead, the reaction resulted in the formation of the Glaser coupling product **4é´** in excellent yield. These results indicate that electronics and substitution patterns of an alkyne substrate can promote distinct reaction pathways with AgF_2_ that yield value-added products.

## Dearomative difluorination of heterocycles:

Having established the ability of AgF_2_ to add fluorine atoms to alkenes and alkynes, we were curious whether these conditions could be used to perform dearomative difluorination of aromatic heterocycles such as indoles and benzofurans. There is an increased interest in converting planar aromatic heterocycles to their 3D analogs based upon correlations observed between the clinical success of drug molecules and the increase in saturation/chiral centers (commonly referred to as Fsp^3^, the number of sp^3^ hybridized carbons/total carbon count in its chemical structure).(18) The favorable effect of increasing the Fsp^3^ of these aromatic scaffolds can be further enhanced if accompanied by the installation of fluorine atoms at the newly generated sp^3^ centers due to the aforementioned benefits of fluorine substitution in enhancing drug efficacy.(19)

While the reactions of unprotected and *N*-methyl protected indoles with AgF_2_ in acetonitrile at room temperature were ineffective, we found that *N*-acyl protected indole (**5a**) delivered 56% isolated yield of the *trans* diastereomer of 2,3-difluoroindolines (**6a**) under these conditions (Fig. 3B). Analysis of the crude reaction mixture indicated that the crude *trans*:*cis* ratio was ~3:1, suggesting that the combined yield of the diastereomers is ~80%. We proceeded to explore a range of substituted indoles (**5c**–**h**), including methyl substitution at the 3-position (**5b**). All substrates gave good yields of the *trans*-diastereomer of the desired product (**6b**-**6h**) with the crude d.r. varying from 2.3:1 and up to 7.3:1. Finally, other heterocycles such as benzofurans (**5i**–**m**) and benzothiophene (**5n**) provide moderate yields of the difluorinated products (**6i**–**n**).

## Fluorination of sp^3^ C-H bonds:

The direct activation and fluorinative functionalization of sp^3^ C-H bonds (aliphatic fluorination) is an area of intense research efforts (20) with seminal contributions from Groves, (21) Leckta,(22, 23) Sarpong,(24) Sorensen,(25) Chen,(26) Tang,(27) and Barnum.(28) This reaction presents unique challenges since it requires the activation of a relatively unreactive C-H bond followed by installation of a fluorine atom. Many of these seminal reports utilize a precious transition metal catalyst to activate the C-H bond and almost all methodologies utilize a separate fluorine source (Selectfluor, NFSI etc.) to install the fluorine in place of the C-H bond. Recognizing the potential of F^•^ to abstract relatively inert C-H bonds (**7**) (due to the favorability of formation of a strong H-F bond), we hypothesized that AgF_2_ in acetonitrile might have the ability to generate a carbon-centered radical. This radical could either directly yield the fluorinated product (**8**) by F-atom transfer from another molecule of AgF_2_ or form an alkyl-Ag(III)F_2_ intermediate followed by reductive elimination to deliver **8**.

**Fig. 4.**
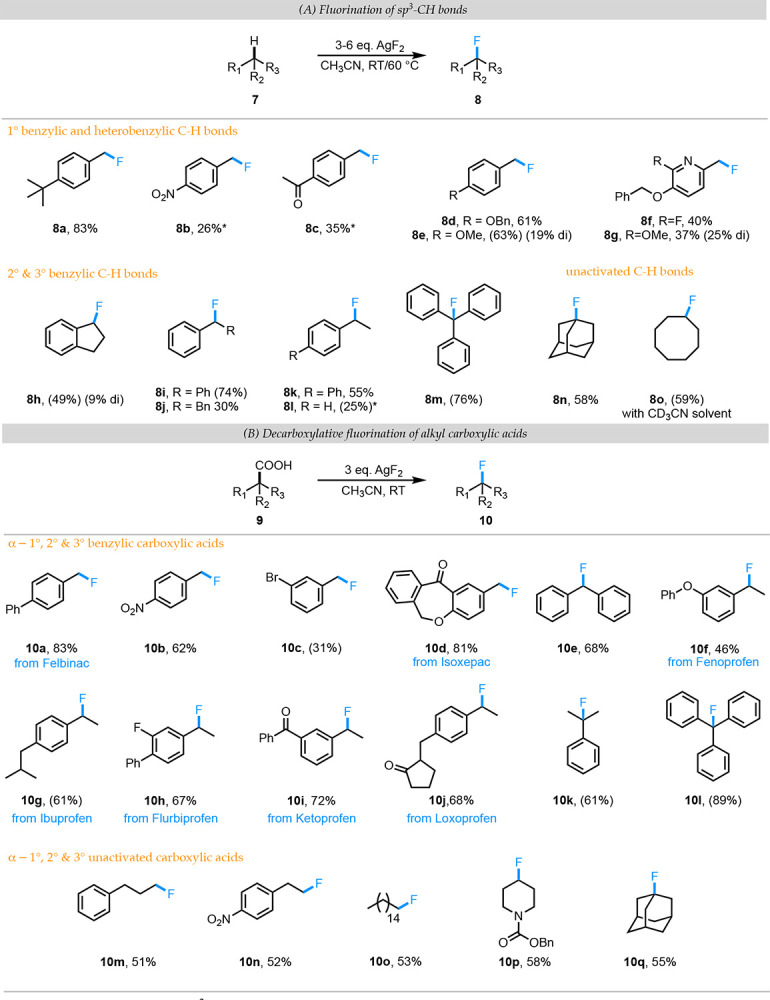
(A) Substrate scope for sp^3^-CH fluorination reaction where AgF2 plays the dual role of C-H activation and C-H functionalization. ^#^5–20% of difluorinated product in these reactions. (B) Substrate scope for decarboxylative fluorination reaction where AgF2 plays the dual role of decarboxylation reagent and fluorine source. Yields in parenthesis are NMR yields based on inert internal standard added to the reaction mixture.* unreacted SM due to solvent fluorination.

We tested our hypothesis that AgF_2_ in acetonitrile might have the ability both activate and functionalize C(sp^3^)–H bonds by using electron-rich toluenes and 3.0 equivalents of AgF_2_ at room temperature. Under these conditions, we obtained ~60–80% yields of the benzylic fluorination product (Fig. 4A, **8a**, **8d**, **8e**, **8g**). The tertiary benzylic C-H bond of triphenyl methane was smoothly fluorinated under these conditions to deliver 76% (NMR yield) of the desired C–H fluorinated **8m**. Electron-deficient toluenes and 2° benzylic C-H bonds required higher temperatures (upto 60 °C) and larger excess of AgF_2_ reagent (~6 equivalents) to get low to moderate yields of the mono-fluorinated products (**8b**, **8c**, **8f**, **8h**-**8l**) – a result that we attribute to competing fluorination of the solvent by AgF_2_. Adamantane was fluorinated in good yield at 70 °C and using 6 equivalents of AgF_2_ to give a single product (**8n**) arising from 3° C-H fluorination. Our attempts to fluorinate other unactivated 2° and 3° C-H bonds under these conditions resulted in only trace amounts of the desired products due to solvent fluorination (See Supporting Information). Switching to dry *d3*-acetonitrile as solvent suppressed solvent fluorination and 59% NMR yield of fluorocyclooctane was obtained at room temperature.

## Decarboxylative fluorination of aliphatic carboxylic acids:

The selective installation of fluorine at specific positions in a molecule is a highly significant transformation. The decarboxylative fluorination of aliphatic carboxylic acids represents a mode where readily available carboxylic acid can undergo site-specific fluorination by replacement of the carboxylic acid group with fluorine. The ability of silver(II) complexes to decarboxylate aliphatic carboxylic acids is well-documented by Anderson and Kochi.(29) This reaction is proposed to form an alkyl radical as a key intermediate upon decarboxylation. In 2012, Li and coworkers reported the silver catalyzed decarboxylative fluorination reaction.(30) In this work, they utilized silver(I) nitrate (AgNO_3_) as catalyst with Selectfluor as the fluorine source under aqueous conditions to form the desired decarboxylative fluorination product. Macmillan,(31) Hu,(32) and Ye(33) have utilized both inorganic complexes and organic molecules as photocatalysts to carry out the photoredox catalyzed decarboxylative fluorination of alkyl carboxylic acids. Once again, an alkyl radical is generated by the decarboxylation of a carboxyl radical, which is formed by oxidation of a carboxylate by the photocatalyst. The alkyl radical is then trapped by either Selectfluor or nucleophilic fluorine sources.

A common feature of these decarboxylative fluorinations is that they utilize a “decarboxylating catalyst” and a “fluorinating reagent”. Based on the established propensity of silver(II) complexes to decarboxylate and the ability of AgF_2_ to fluorinate transient alkyl radicals, we questioned whether AgF_2_ in acetonitrile could be a unique reagent that can carry out both steps in a decarboxylative fluorination reaction; eliminating the need for expensive photocatalysts, added bases, or specialized fluorinating reagents. Hartwig and co-workers utilized this strategy to synthesize trifluoromethyl ethers from α-phenoxy-α,α-difluoro acetic acid.(34) However, they did not report a more general analogous reaction with simple carboxylic acids.

We initiated our exploration of this simple protocol for decarboxylative fluorination by subjecting a range of 1° (**9a**-**d**), 2° (**9e**-**j**), and 3° (**9k**-**l**) α-benzylic carboxylic acids to 3 equivalents of AgF_2_ in acetonitrile at room temperature (Fig. 4B). In validation of our hypothesis, the prod ucts of decarboxylative fluorination (**10a**-**l**) were obtained in >60% yield for nearly all these substrates. Several of these substrates are commercially available NSAIDs which have other functional groups in addition to the alkyl carboxylic acid thus highlighting the remarkable chemoselectivity of the decarboxylative fluorination reaction. To further test the generality of this protocol, we also subjected a few unactivated α−1°, 2°, and 3° alkyl carboxylic acids (**9m**-**q**) to these conditions. In each of these cases, the desired product (**10m**-**q**) was obtained in 50–60% isolated yield including substrates with potentially reactive C-H bonds such as *N*-protected piperidine 4-carboxylic acid (**9p**) and adamantyl carboxylic acid (**10q**). To our knowledge, this represents the most straightforward and robust protocol for this important transformation.

## Mechanistic experiments:

**Fig. 5.**
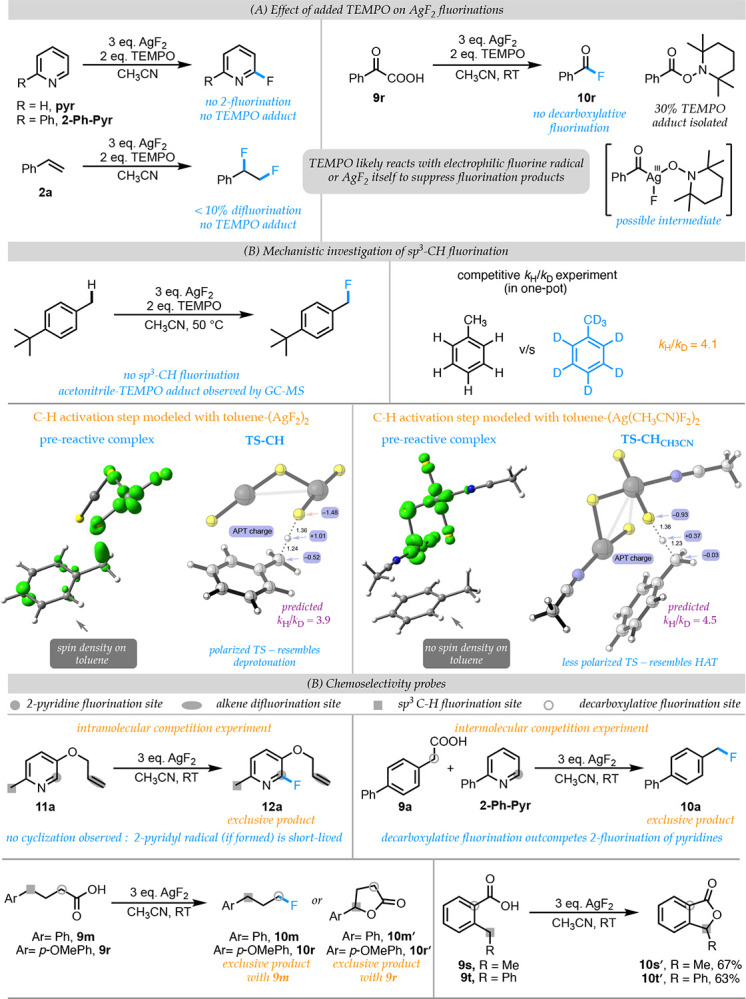
(A) Experiments with added TEMPO to probe for potential radical intermediates in these reactions. (B) KIE and DFT studies for the sp^3^-CH fluorination chemistry that highlights role of acetonitrile in facilitating radical fluorine chemistry. (C) Evaluation of chemoselectivity in reactants with multiple potential fluorination sites.

To probe the intermediacy of radical species in AgF_2_ mediated pyridine fluorination and alkene difluorination reactions, we performed reactions of pyridine, 2-phenylpyridine and styrene in the presence of 2 equivalents of TEMPO. All three reactions were inhibited by TEMPO with pyridine and 2-phenylpyridine yielding none of the desired pyridine fluorination product and styrene yielding only ~5–7 % of the expected difluorination product. We were unable to isolate any TEMPO adducts in these reactions and the unreacted starting materials could be observed by GC-MS analysis of the crude reaction mixture. This suggests that TEMPO might be reacting with either F^•^ or AgF_2_ to suppress reactivity (Fig. 5A, left panel). Next, we subjected phenyl glyoxylic acid to the standard decarboxylative fluorination conditions both with and without added TEMPO. In the absence of TEMPO, we obtained excellent NMR yields of benzoyl fluoride. In the presence of 2 equivalents of TEMPO, none of the benzoyl fluoride product was formed and instead ~30% of the TEMPO adduct was isolated. We interpret this result as evidence for trapping of a persistent benzaldehyde radical formed via decarboxylation of a Ag(II) salt of phenyl glyoxylate in a mechanism analogous to that proposed by Anderson and Kochi(29) (Fig. 5A, right panel). Addition of 2 equivalents of TEMPO to the reaction of 4-*t*-butyl toluene at 50 °C resulted in complete suppression of the sp^3^-CH fluorination product. Interestingly, at this elevated temperature, GC-MS analysis of the reaction mixture showed formation of significant amounts of the TEMPO adduct with acetonitrile radical (Fig. 5B).

To gain further insight into the mechanism of sp^3^ C-H fluorination, we performed a competitive experiment with a 50:50 mixture of toluene:*d-8* toluene and obtained a *k*_H_/*k*_D_ of 4.1 indicating that C-H bond-cleavage occurs in the rate-determining step (Fig. 5B). For the quantitative interpretation of this KIE, we modeled the transition state for C-H bond-cleavage of toluene by (AgF_2_)_2,_ with and without solvent coordination. In the model without acetonitrile as ligand, analysis of the pre-reactive complex of (AgF_2_)_2_ and toluene reveals significant spin-density in the aromatic ring (Fig. 5B). This is suggestive of an oxidative mechanism involving partial transfer of electron density from toluene to (AgF_2_)_2_ *prior* to C-H bond-cleavage. Consequently, the transition state for C-H bond cleavage (Fig. 5B, **TS-CH**) is quite polarized with the hydrogen being transferred as a proton (see APT charges). In contrast, analysis of the pre-reactive complex of (AgF_2_)_2_ and toluene with acetonitrile as ligand shows little to no spin density on the aromatic ring and the resulting transition state (Fig. 5B, **TS-CHCH3CN**) resembles an H-atom transfer. This is consistent with our initial finding that acetonitrile coordination makes silver (II) fluoride a milder oxidant and facilitates radical reactivity. The predicted *k*_H_/*k*_D_ for both TSs are in good agreement with the experimental *k*_H_/*k*_D_ (Fig. 5B).

We also designed probes to evaluate the chemoselectivity of our fluorination protocol when multiple potential reaction sites are present (Fig. 5C). We subjected 2-methyl-5-allyloxy pyridine (**11a**) with three potential reaction sites (2-pyridyl, benzylic C-H, alkene) to standard reaction conditions and observed pyridine fluorination as the exclusive product (**12a**). This suggests that formation of **pyr-(AgF**_**2**_**)**_**2**_ complex dictates the chemoselectivity of this reaction. We also note the absence of any cyclization products from the trapping of the putative 2-pyridyl radical by the pendent allyloxy group of **11a** – an observation that indicates that the 2-pyridyl radical, if formed, has a fleeting existence and is likely rapidly captured by silver as proposed in our initial computational study (Fig. 2A). Next, we subjected an equimolar mixture of **9a** and 2-phenylpyridine to our fluorination conditions and observed exclusive formation of the decarboxylative fluorination product **10a**. This result suggests that the carboxylic acid likely binds stronger to AgF_2_ than the pyridine nitrogen. Finally, we subjected 4-aryl butanoic acids (**9m** and **9r**) to decarboxylative fluorination conditions; while the 4-phenyl butanoic acid **9m** allows exclusive formation of the expected product **10m**, 4-*p*-methoxy butanoic acid (**9r**) gave none of the expected product **10r** but instead exclusively formed the 5-membered lactone product **10ŕ**. Presumably, the more activated benzylic C-H bonds of **9r** facilitates C-H functionalization over decarboxylative fluorination. Formation of the lactone **10ŕ** suggests a mechanism involving C-O bond-forming reductive elimination from a Ag(III) intermediate. We were able to extend this lactonization reaction to 2-ethyl benzoic acid (**9s**), an aromatic acid that is not a viable substrate for the decarboxylative fluorination reaction. We isolated the lactone product **10ś** exclusively from this reaction, suggesting that the carboxylic acid group of benzoic acids could enable directed sp^3^-CH lactonization.

In conclusion, we have disclosed a novel platform for the generation of the highly reactive fluorine radical for applications in C–F bond-forming reactions. This platform delivers valuable organofluorine compounds using a single reagent under mild conditions. The excellent chemoselectivity of this reaction suggests that this technology will find utility in the late-stage fluorination of drug molecules, thus enabling rapid access to important SAR studies. Detailed mechanistic studies of the methodology reported in this study will direct the discovery of new reactivity including development of catalytic variants that can leverage the unique reactivity of silver (II) fluoride complexes.

## Supplementary Material

Supplementary Files

This is a list of supplementary files associated with this preprint. Click to download.

- 0725SI.pdf
